# Dissecting Latency in 360° Video Camera Sensing Systems †

**DOI:** 10.3390/s22166001

**Published:** 2022-08-11

**Authors:** Zhisheng Yan, Jun Yi

**Affiliations:** 1 Department of Information Sciences and Technology, School of Computing, George Mason University, Fairfax, VA 22030, USA; 2 Department of Computer Science, Georgia State University, Atlanta, GA 30302, USA

**Keywords:** 360° video camera sensing, latency, measurement study, wireless multimedia sensor networks

## Abstract

360° video camera sensing is an increasingly popular technology. Compared with traditional 2D video systems, it is challenging to ensure the viewing experience in 360° video camera sensing because the massive omnidirectional data introduce adverse effects on start-up delay, event-to-eye delay, and frame rate. Therefore, understanding the time consumption of computing tasks in 360° video camera sensing becomes the prerequisite to improving the system’s delay performance and viewing experience. Despite the prior measurement studies on 360° video systems, none of them delves into the system pipeline and dissects the latency at the task level. In this paper, we perform the first in-depth measurement study of task-level time consumption for 360° video camera sensing. We start with identifying the subtle relationship between the three delay metrics and the time consumption breakdown across the system computing task. Next, we develop an open research prototype Zeus to characterize this relationship in various realistic usage scenarios. Our measurement of task-level time consumption demonstrates the importance of the camera CPU-GPU transfer and the server initialization, as well as the negligible effect of 360° video stitching on the delay metrics. Finally, we compare Zeus with a commercial system to validate that our results are representative and can be used to improve today’s 360° video camera sensing systems.

## 1. Introduction

Camera sensing through visual sensors has been an important technology in wireless sensor networks [1,2,3]. Recently, there has been an increasing interest in 360° cameras. Compared with traditional cameras, the 360° camera is an emerging visual sensor that can record a remote scene in all directions. By live-streaming the omnidirectional visuals to distant viewers through networks, 360° video camera sensing systems are able to offer an immersive viewing experience, where viewers can enjoy the complete field-of-view of a remote site. This enables a broad spectrum of new applications such as panoramic wildlife monitoring in scientific studies, all-around security surveillance in airports, and 360° video Internet viewing for entertainment [4].

A critical requirement of 360° video camera sensing systems is the delay because it plays a pivotal role in ensuring users’ viewing experience. Complex system initialization may lead to an excessive *start-up delay*, decreasing users’ willingness to continue the viewing. The start-up delay may in turn incur a long *event-to-eye delay* which is the time interval between when an event occurs on the remote scene and when it is displayed on the user’s screen. Long event-to-eye delay causes significant lags in streaming live events such as sports and business meetings and would be detrimental in applications that require real-time interaction. Moreover, the *frame rate* of the 360° video is determined by how fast frames can be pushed through the system pipeline. A low frame rate would lead to an unappealing viewing experience for the users.

It is challenging to guarantee acceptable delay and user experience in 360° video camera sensing systems. This is because 360° video camera sensing systems generate far more data than traditional 2D video camera sensing systems. A fundamental prerequisite to preventing undesirable user experience caused by delays is understanding how different components of a 360° video camera sensing system contribute to the aforementioned three delay metrics.

Although many measurement studies have been conducted on 2D video camera sensing and streaming [5,6,7], the delay of 360° video camera sensing systems has not been well understood. Recent studies in 360° videos focused on rate-adaptive streaming [8,9,10,11] and video encoding/projection [12,13,14]. The only two existing measurement studies on live 360° video systems [15,16] only treated the system as a black box and performed system-level measurements. For example, our previous study on YouTube live streaming services [15] showed that the event-to-eye delay is unacceptably large. Users need to wait for up to 42 s before viewing the most recent events captured by remote cameras. Although these early works identified the problem of delay and the dissatisfaction of users in 360° video camera sensing, they did not pinpoint the underlying limitations of today’s 360° video camera sensing systems across the system design pipeline. Specifically, they were not able to dissect the sensing and streaming pipeline to examine how each task of a 360° video camera sensing system contributes to the start-up delay, event-to-eye delay, and frame rate. As a result, the bottlenecks of the 360° video camera sensing are still unknown, making it difficult to optimize the system performance and improving the user experience.

The goal of this paper is to fill this gap and conduct an in-depth measurement study of the time consumption across the end-to-end system pipeline in 360° video camera sensing. We aim to identify the bottleneck of a 360° video camera sensing system in terms of different delay metrics and call for design guidelines for optimized 360° video systems.

One key challenge of the proposed measurement is that commercial 360° video systems usually behave as black boxes. The closed-source nature of these systems makes it almost impossible to study the per-task delay of the 360° video camera sensing pipeline. In this paper, we overcome this challenge by building a 360° video camera sensing research prototype—Zeus. Zeus includes a 360° camera, camera-server transmission, a video server, server-client transmission, and a video client. Implemented by off-the-shelf hardware, publicly available SDKs, and open-source software packages, Zeus can be easily reproduced for future camera sensing research areas, e.g., simulation, modeling, and algorithm design.

We measure the time consumption of each computing task in all five system components on Zeus and profile the delay characteristics of the system under various realistic scenarios. Our micro- and macro-benchmarks demonstrate four crucial findings. First, transferring video frames between the CPU and GPU in the camera is a critical task for reaching a satisfactory frame rate because this internal camera transfer consumes non-negligible time. Second, stitching a 360° video frame surprisingly has a negligible effect on the frame rate. Third, server initialization before the live video streaming is greatly time-consuming. This long start-up delay adds to a significant event-to-eye delay, which could lead to an annoying time lag between what happens in the remote scene and what is displayed on the screen. Fourth, we found across different real-world scenarios that camera motion is a deciding factor in the system delay and viewing experience. Overall, the 360° camera is the bottleneck for frame rate, whereas the server is the primary cause for low start-up and event-to-eye delay.

As the implementations of Zeus and commercial 360° video platforms are not exactly the same, the absolute values of the results obtained with Zeus may be different from those measured on commercial platforms. Hence, we additionally measure the delay on a commercial system that is based on Ricoh Theta V 360° camera and YouTube live streaming servers. We treat it as a black box and compare its component-wise time consumption with Zeus. The comparison shows a strong correlation between the two systems, indicating that our observations can be generalized to exiting 360° video camera sensing systems in the current practice.

In sum, the contributions of the paper are

- We identify the relationship between the three delay metrics and the time consumption breakdown across the system computing tasks (Section 3.2).
- We launch an open research prototype Zeus (https://github.com/junyiwo/Zeus (accessed on 27 June 2022)). The methodology for building Zeus can be reused in future 360° video research (Section 3.3).
- We perform an in-depth delay measurement to dissect the time consumption in 360° video camera sensing and compare Zeus against a commercial live 360° video system to validate our findings (Section 4 and Section 5).

## 2. Related Work

### 2.1. 2D Video-on-Demand Streaming Systems

Video-on-demand (VoD) streaming services over Internet such as YouTube and Vimeo were widely examined [17,18,19,20,21,22]. Different from video camera sensing systems, videos are pre-stored at VoD servers and then streamed to users. No live camera capturing is involved. Different metrics for quality of experience (QoE) of 2D VoD streaming services have been measured by various tools [17]. For example, a large-scale study of YouTube video streaming over IPv6 and IPv4 [18] showed that the playback stall is a more serious problem in IPv6 networks. A comprehensive analysis of 12 common mobile VoD streaming services over cellular networks [20] found that two to three segments are the ideal startup buffer size to reduce the possibility of playback stall. While these measurements provide insight into video streaming performance, the methodology cannot be directly used in video camera sensing systems that have a different system pipeline.

### 2.2. 360° VoD Streaming Systems

Measurement studies on streaming pre-recorded 360° videos have received a growing interest [14,23,24,25,26,27]. Researchers investigated thousands of pre-recorded 360° videos on YouTube across more than a dozen categories and found that delay is a key criterion when streaming and viewing 360° videos in VR headsets [23]. The video encoding and streaming methods of Oculus 360° VoD streaming were studied in [14]. The authors reverse-engineered the offset cubic projection advocated by Oculus. This popular project method encodes a distorted version of the spherical surface and assigns more bits to a particular view of the cubic. Other measurement studies discovered that the impacts of delay on 360° VoD streaming mainly stem from the impacts of delay on viewport-adaptive streaming algorithms [24,25,28]. Despite all the efforts to understand 360° VoD systems, none considers the 360° camera and the management of a live streaming session, which are essential components in 360° video camera sensing.

### 2.3. 2D Video Camera Sensing Systems

A wealth of studies were conducted to understand 2D video camera sensing systems that live capture and stream 2D videos. The delay analysis of Periscope, a popular live video App, showed that client buffering, chunking, and polling are the major contributors to the high latency of the HTTP-based live streaming systems [5]. The QoE for camera sensing and live video on mobile devices was also studies [6]. The observations indicated that live video streaming time is significantly affected by the network protocols used for streaming. Other measurement studies [29,30] investigated encoding algorithms to make them bandwidth-efficient in order to reduce the live video streaming time. Although this line of research is impactful on video camera sensing system design, the observations cannot be applied to 360° videos since more than one video views and additional computing tasks are enforced in 360° video camera sensing.

### 2.4. 360° Video Camera Sensing Systems

A limited number of studies were focused on 360° video camera sensing. 360° video camera sensing using commercial cameras and YouTube live streaming platforms was investigated recently. The results for streaming videos with up to 4 K resolution showed that users suffer from a high event-to-eye delay in live 360° video systems [15]. Similarly, a crowd-sourced study on YouTube and Facebook streaming platforms using simulated live camera streams was conducted [16]. The measurement results confirmed the high event-to-eye delay and demonstrated that users also experience long playback stalls. Chen et al. [11] proposed a 360° video stitching method to ensure the delay in tile-based live 360° video streaming under strict time budgets. Despite the improved understanding of commercial live 360° video streaming platforms, none of the existing studies probes into the camera performance. Moreover, existing works do not dissect the delay of a 360° video camera sensing pipeline at the component or task level and fail to discover the impacts of components/tasks on delay metrics (start-up, event-to-eye, and frame rate).

In contrast, the proposed study delves into each component of a canonical 360° video camera sensing system and each task of the components in order to perform the in-depth delay analysis. Our preliminary results were shown in [31]. This paper takes a step further and expands the measurement from one experiment setup to five usage scenarios by varying the camera motion and the sensing location. Additional validation of our results against commercial platforms is also included. Our extensive results confirm the generality of the measurement findings.

## 3. Measurement Design

In this section, we introduce the design of our measurement study including system architecture, delay metrics, and Zeus prototype. We also explain the rationale of our measurement design in dissecting the latency of 360° video camera sensing.

### 3.1. Identifying Canonical System Architecture

**Figure 1:**
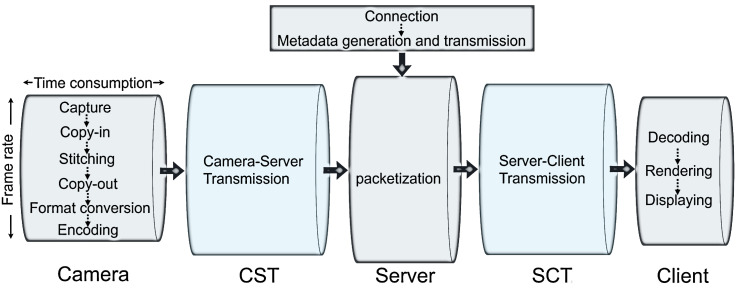
The architecture of 360° video camera sensing and the tasks of the 5 system components. The top rectangle represents one-time tasks whereas the 5 bottom pipes are the pipeline tasks that must be passed through for every frame.

We first discuss the canonical system architecture of 360° video camera sensing as illustrated in Figure 1. The computing pipeline of 360° video camera sensing consists of five *components*—a camera, camera-server transmission (CST), a server, server-client transmission (SCT), and a client. Each component must complete several computing *tasks* in sequence.

In particular, the 360° camera is typically composed of multiple 2D camera lenses configured in specific directions to cover the ominidirectional scene. Multiple 2D video frames of a remote scene are first captured. These frames are moved to the camera GPU and stitched into a 360° equirectangular video frame. The equirectangular frame is transferred to the camera CPU and then encoded at the CPU. The camera then uploads the frame to the server via its wireless connection to the Internet. The server parses the video data received from the camera and stores it in a video buffer in the server memory. Before the buffered video data reaches a certain amount, no client request will be accepted by the server. Once the buffered video data is sufficient, a URL to access the live streaming session will become available. Clients (e.g., PCs, head-mounted displays, and smartphones) can then initiate live streaming via the URL. After a connection between the client and the server is established, the server creates a metadata file about the streaming session and sends it to the client. Then the server starts streaming data from its buffer. Upon receiving data packets from the server, the client decodes, renders, and displays 360° video frames on the screen.

Table 1 summarizes these computing tasks across system components. It should be emphasized that the connection and metadata generation are *one-time tasks* for a given session between the server and the client, whereas all other tasks are *pipeline tasks* that must be passed through for every video frame. We aim to measure the delay of all these tasks.

### 3.2. Analyzing Delay Metrics

We now identify the delay metrics that affect viewing experience and discuss how they are determined by the time consumption of system components. The amount of time consumption is denoted by the length of a pipe in Figure 1.

#### 3.2.1. Start-Up Delay

Start-up delay defines the time it takes for the first video frame to display on the client screen since a client initiates a viewing session. One main reason discouraging users from continuing and complete the video viewing is the long start-up delay [32]. We formally denote the time consumption for the one-time connection and metadata generation as $T_{srv,once}$, the server-client transmission of a frame as $T_{sct}$, and the processing and display of a frame on the client device as $T_{clnt}$. We then express the start-up delay $D_{start}$, i.e.,

(1) $$D_{start}=T_{srv,once}+T_{sct}+T_{clnt}.$$

Note that the time consumption in the camera and camera-server transmission does not affect the start-up delay. The reason is that the live streaming of camera-recorded videos will not start until the server buffers sufficient video frames from the camera. Hence, video frames should have already been stored in the server before the server creates the streaming URL and accepts the client’s request.

#### 3.2.2. Event-to-Eye Delay

The time difference between the moment when an event occurs on the remote scene and the moment when the event is displayed on the user screen is the event-to-eye delay. An excessive event-to-eye delay makes users perceive the annoying mismatch and lag between what happens and what is displayed in camera sensing of live events and activities. This greatly degrades the responsiveness of real-time communications in interactive applications such as disaster command and response [33]. The event-to-eye delay $D_{event-to-eye}$ is determined by all computing tasks in 360° video camera sensing. Video frames must spend time at all components and undergo each task before being displayed, i.e.,

(2) $$\begin{array}{c} D_{event-to-eye}=T_{cam}+T_{cst}+T_{srv,once}+T_{srv,pipe}+T_{sct}+T_{clnt} \end{array}$$

where $T_{cam}$ is the time a frame spends on the camera, $T_{cst}$ is the camera-server transmission time, and $T_{srv,pipe}$ is the time consumption of a frame going through the pipeline tasks in the server (packetization). We note that although not all frames experience one-time connection and metadata tasks, their time consumption is propagated to subsequent frames, thus contributing to the event-to-eye delay.

#### 3.2.3. Frame Rate

Frame rate represents the number of frames that can be processed and pushed through system components in the pipeline per unit time. The end-to-end frame rate of the system, FR, must be greater than a threshold to ensure the smooth video playback on the client screen. By considering the system as a set of pipes, the system frame rate is jointly determined by the frame rates of all system components (all individual pipes) and can be defined by the minimum of these frame rates (the narrowest pipe). Formally, we have

(3) $$FR=\min\{FR_{cam},FR_{cst},FR_{srv},FR_{sct},FR_{clnt}\}$$

where $FR_{cam},FR_{cst},FR_{srv},FR_{sct},FR_{clnt}$ are the frame rate of each system component. It is important to note that the frame rate of a component, i.e., how many frames can flow through a pipe per unit time, is not necessarily the inverse of the per-frame time consumption on that component. Only if the tasks within the component are run sequentially, e.g., in the camera, the frame rate is the inverse of the per-frame time consumption. If tasks in that component are executed in parallel by different hardware units, e.g., in the server, this rule does not hold. As shown in Figure 1, the end-to-end frame rate is determined by the radius rather than the length of each pipe. We will scrutinize how tasks affect frame rate in Section 4.

#### 3.2.4. Delay Dissection at the Task Level

We have defined the three delay metrics in terms of the time consumption of system components. It is straightforward to dissect the delay metrics using task-level time consumption because the tasks within each component are mostly serialized. Specifically, $T_{cam},T_{srv,once},T_{srv,pipe},T_{clnt}$ can be represented as follows.  

(4) $$\begin{array}{cc} \; & T_{cam}=T_{capt}+T_{cpin}+T_{stch}+T_{cpout}+T_{cnvt}+T_{encode}\\ \; & T_{srv,once}=T_{connect}+T_{meta}\\ \; & T_{srv,pipe}=T_{pkt}\\ \; & T_{clnt}=T_{decode}+T_{rndr}+T_{dsply} \end{array}$$

By replacing the component time in (1)–(3) by the above Equation (4), we can dissect the delay metrics at the task level.

### 3.3. Implementing Zeus Prototype

**Figure 2:**
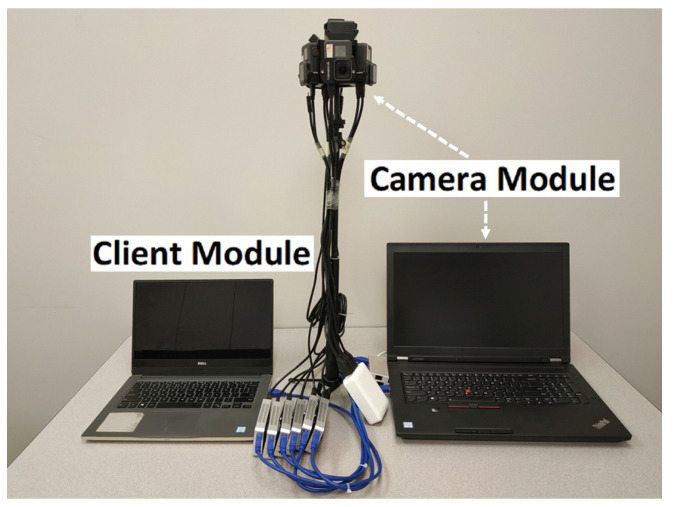
The Zeus prototype.

Measuring the delay in 360° video camera sensing systems is challenging because camera sensing systems using commercial cameras and live streaming platforms are closed-source. Furthermore, no measurement tool is available to examine the black-box commercial cameras (e.g., Ricoh Theta V), live streaming servers (e.g., Facebook), and players (e.g., YouTube). Although commercial APIs are provided to allow App development, none of them can be used to obtain the delay breakdown at the task level. To enable the measurement of how the time consumption at the task level affects 360° video camera sensing and user experience, we build an end-to-end system prototype, Zeus, shown in Figure 2, as a reference implementation to the canonical architecture. We implement Zeus by only using off-the-shelf hardware, publicly available SDKs, and open-source software packages such that the research community can readily reproduce the reference implementation for future research.

#### 3.3.1. Hardware Design

Similar to a commercial 360° camera, the 360° camera in Zeus consists of multiple 2D camera lenses—six GoPro Hero cameras [34]. These 2D cameras are held and positioned by a 360° camera rig and connected to a laptop that serves as the processing unit. The 2D camera visuals are processed by six HDMI capture cards and then combined and fed to the processing unit via three USB 3.0 hubs. The laptop processing unit has an 8-core CPU at 3.1 GHz and an NVIDIA Quadro P4000 GPU. The processing power makes it feasible to process, stitch, and encode 360° videos. The video server is deployed at a university data center which runs Ubuntu 18.04.3 LTS. The client device is a laptop running Windows 10 with an Intel Core i7-6600U CPU and an integrated HD graphics card.

#### 3.3.2. Software Design

To capture wide-angle video frames from the 2D camera lenses, we configure the six GoPro in the SuperView mode. The VRWorks 360 Video SDK [35] is used to move, store, and stitch the 2D video frames. One of the primary challenges in the camera design is the 360° video stitching quality. We first calculate camera distortion parameters [36] using the OpenCV function cv.fisheye.ca- librate() and the second-order distortion model [37] to reduce the effects of camera lens distortion during stitching. We then utilize these camera parameters to calibrate the video frames during stitching. This calibration ensures that the overlapping areas of two adjacent frames will not be distorted. A sample configuration file recording the camera resolution and the size and position of the camera rig is shown in Listing 1 and is used as input to the VRWorks 360 Video SDK. The 2D frames are transferred to the GPU via cudaMemcpy2D() and stitched to a 360° frame via nvssVideoStitch(). Finally, FFmpeg is used for encoding and streaming the 360° video. Real-Time Message Protocol (RTMP) is utilized to upload the 360° video to the server for low-delay transmission. Our upload protocol is similar to most commercial cameras, e.g., Ricoh Theta V and Samsung Gear 360.

Listing 1 **XML file of the camera rig.**

<?xml version="1.0" encoding="utf-8"?>

<rig coord_axes="z-up" rig_diameter_cm="9.5">

<camera width="1024" height="768" layout="equatorial">

<input_calib_file name="camera1.jpg"/>

<pose>

<rotation yaw_deg ="0" pitch_deg ="0.0" roll_deg="-90.0" />

......

</rig>

As for the video server, we run an open-source Nginx-1.16.1 web server. The video is streamed from the server to the client through the HTTP-FLV protocol since HTTP-FLV can penetrate firewalls and is more acceptable to web servers. While other popular live streaming protocols, e.g., HLS, may have been used, HLS protocol consumes significant time for segmenting a video stream into chunks with different video quality. As a result, the start-up delay might be higher. To allow the server to receive RTMP live video streams from the 360° camera and deliver HTTP-FLV streams to the client, we configure Nginx as the *nginx-http-flv-module* [38].

To support the general applicability of client software, the client is implemented as an HTML5-based web video player using FLV.js, a flash-based module written in JavaScript. We use Three.js to fetch video streams from Flv.js and project the equirectangular frame onto the sphere format using render(). The spherical frame is stored at the HTML5 element <canvas> to display on webpages. The client software is embedded in a Microsoft Edge browser. We enable hardware acceleration to support the rendering and decoding tasks.

#### 3.3.3. Measurement Methodology

We are able to measure the time consumption of most computing tasks by inserting timestamps in Zeus and calculating the time interval before and after certain code functions are executed. The only exceptions are the camera-server transmission (CST) and server-client transmission (SCT), where the video stream is fragmented into packets for transmission because both the RTMP and HTTP protocols are built on top of TCP. Since the frame ID is not visible at the packet level, we cannot measure the actual transmission time of each frame directly and individually. Instead, we propose to approximate this per-frame transmission delay as the average time consumption for transmitting a video frame during the CST and SCT. For example, to derive the per-frame time consumption of CST, we first obtain the time interval between the moment when the camera starts sending the first frame via stream_frame() and the moment when the server stops receiving video data in ngx_rtmp_live_av(). We then divide this time interval by the number of frames transmitted to calculate the average time for transmitting a frame.

## 4. Results

We present the measurement results in this section. Specifically, we show the time consumption of the tasks in 360° video camera sensing under different impact factors. We also repeat the delay measurement using multiple real-world scenarios. For each system component, we discuss how the start-up delay, event-to-eye delay, and frame rate would be affected by system parameters. We aim to identify the potential mitigation of delay issues to improve user experience.

### 4.1. Default Experiment Scenario

Our default measurement is conducted in a typical lab environment located in a university building. The lab hosts the camera and the client. To mimic the real-world conditions experienced by commercial 360° video systems, we deploy the server at another university campus over 800 miles away. Note that putting the camera and the client in the same building does not affect the results because the video data always flows from the camera to the server and then to the client. It is the server location that determines the transmission distance.

We fix the camera on a table so that the video content generally contains desks, office supplies, and researchers. By default, the capture resolution of the 2D cameras is 720p and the stitched 360° video is encoded at 2 Mbps with the resolution ranging from 720p to 1440p (2 K). We do not choose 4 K or larger resolutions for encoding 360° videos because our previous study shows that 4 K live 360° video streaming often leads to poor system performance and user experiences [15]. Since nearly all computing tasks across the system pipeline would be overwhelmed by the massive 4 K data, 4 K 360° videos are not ideal for the goals of identifying system bottlenecks in this research. When streaming the video, we use a constant resolution during a session and do not employ adaptive streaming since we aim to concentrate on the most fundamental pipeline of 360° video streaming without advanced designs. We set the frame rate as 30 frames per second (fps) and the Group of Pictures (GOP) value of the H.264 encoder as 30. The stitched 360° video is uploaded from the camera to the server and then downloaded from the server to the client through the university WiFi with the upload and download bandwidth being 16 Mbps and 20 Mbps, respectively. For each session, we continue the 360° video camera sensing for 2 min and repeat it 20 times. The average and standard deviation of the 20 trials are reported.

### 4.2. 360° camera

#### 4.2.1. Video Capture Task

**Figure 3:**
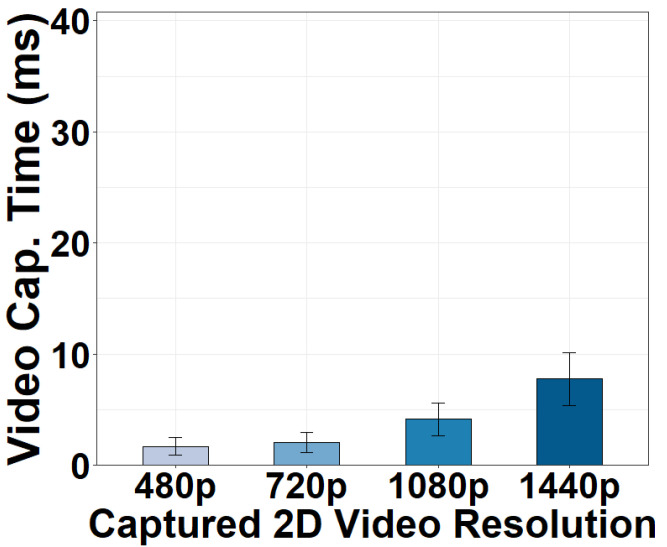
Video capture time versus capture resolutions.

We vary the resolutions of the captured 2D videos and illustrate the video capture time in Figure 3. Compared to the acceptable frame processing delay of a 24–30 fps video (33.3 ms), the video capture time is generally short. Capturing six 480p video frames takes 1.68 ms. The time becomes 2.05 ms for six 720p frames. Both capture resolutions provide abundant details for stitching and are sufficient to generate high-quality 360° videos ranging from 720p to 1440p that are currently supported in today’s 360° video platforms [39,40]. It is true that it spends more time capturing 1080p and 1440p frames. However, capturing such high resolutions of 2D frames for 360° video stitching is typically not required in current 360° video applications.

#### 4.2.2. Copy-In and Copy-Out Tasks

**Figure 4:**
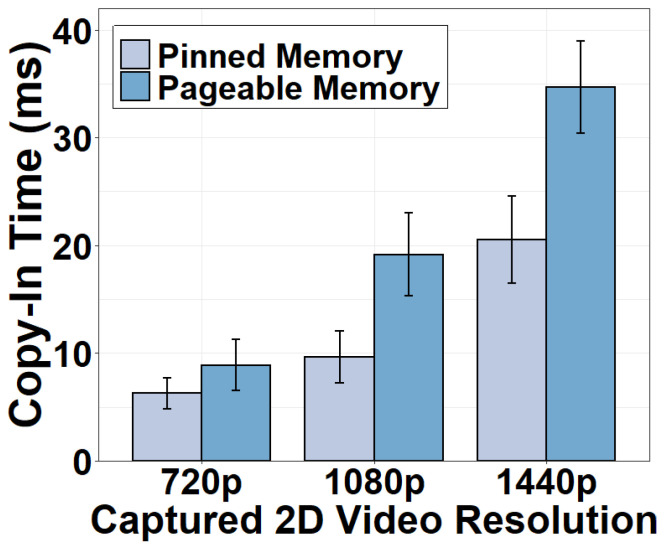
Copy-in time using different memory types.

**Figure 5:**
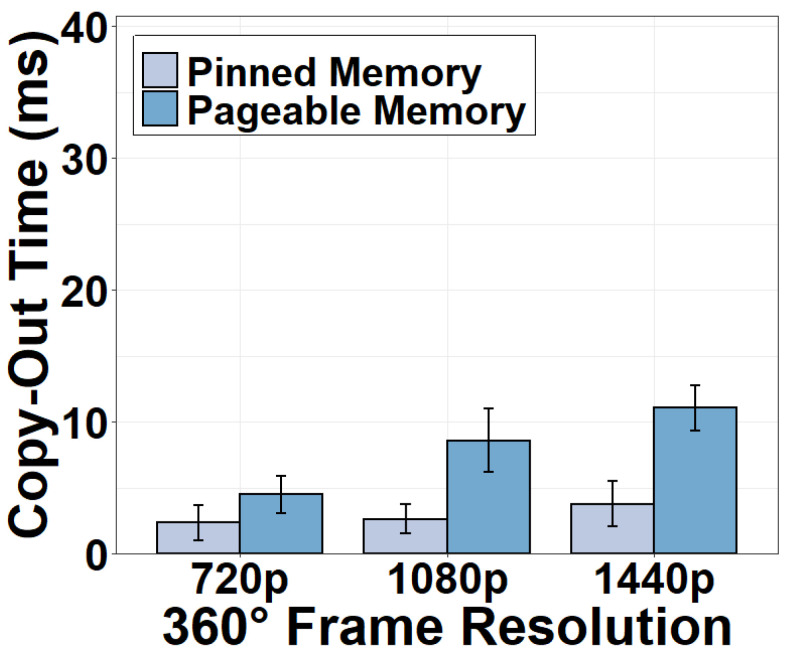
Copy-out time using different memory types.

We next measure the CPU-GPU transfer time using different types of memory. Results in Figure 4 and Figure 5 indicate that the CPU-GPU transfer time is non-negligible. Moving frames from pinned memory to GPU for stitching consumes 6.28 ms for six 720p frames and as high as 20.51 ms for 1440p frames. The copy-out time moving the stitched frame back to the CPU is shorter, consuming 2.33 ms for a 720p 360° frame using the pinned memory and 4.47 ms using the pageable memory. The copy-out is faster because the amount of video data to transfer is smaller after six 2D frames have been stitched as one 360° frame. We conclude that GPU stitching does incur additional overhead. This strategy can be justified only if the benefits of the GPU stitching are superior. It is also clear that the pinned memory is preferred during the CPU-GPU transfer. Pinned memory can directly communicate with the GPU whereas pageable memory has to transfer data between the GPU and the CPU via the pinned memory.

#### 4.2.3. Stitching Task

**Figure 6:**
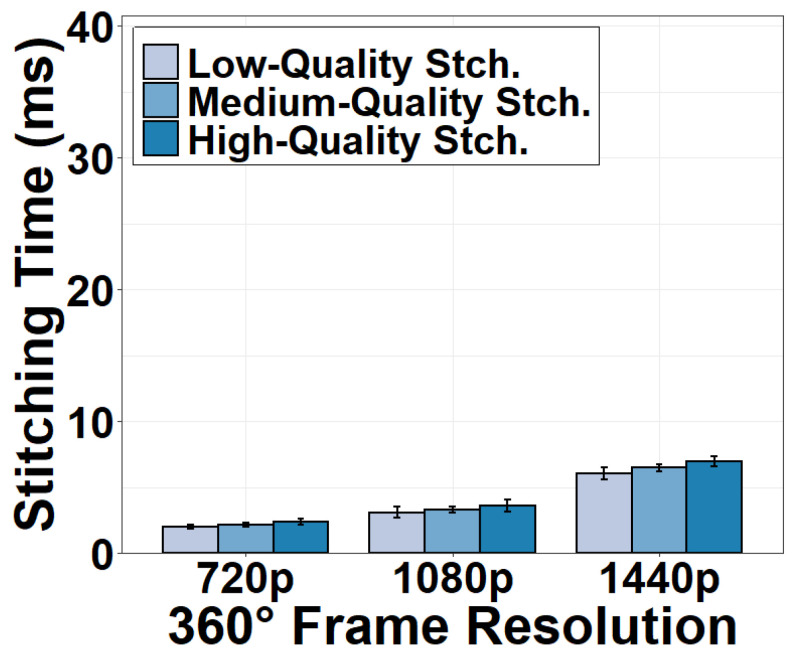
Frame stitching time versus stitching options.

We measure the stitching time under different stitching algorithms. To this end, we configure different levels of stitching quality available in the VRWorks 360 Video SDK to test different algorithms. For instance, “high stitching quality” utilizes a depth-based mono stitching to improve the stitching quality and stability. We discover in Figure 6 that stitching time is surprisingly not a time-sensitive task compared to the CPU-GPU transfer. It takes as low as 1.98 ms to stitch a 720p equirectangular 360° frame in high stitching quality. The stitching time for a 1440p frame is 6.98 ms. Our observation is in sharp contrast to prior 360° video research [41,42] that emphasized the time complexity of 360° video stitching. The short stitching time can be explained as follows. Thanks to the fixed positions of six 2D cameras, today’s GPUs and SDKs can extract the correspondence points between two adjacent 2D frames. They can reuse this correspondence for stitching every frame without having to recalculate the overlapping areas as is done in traditional stitching methods.

#### 4.2.4. Format Conversion Task

**Figure 7:**
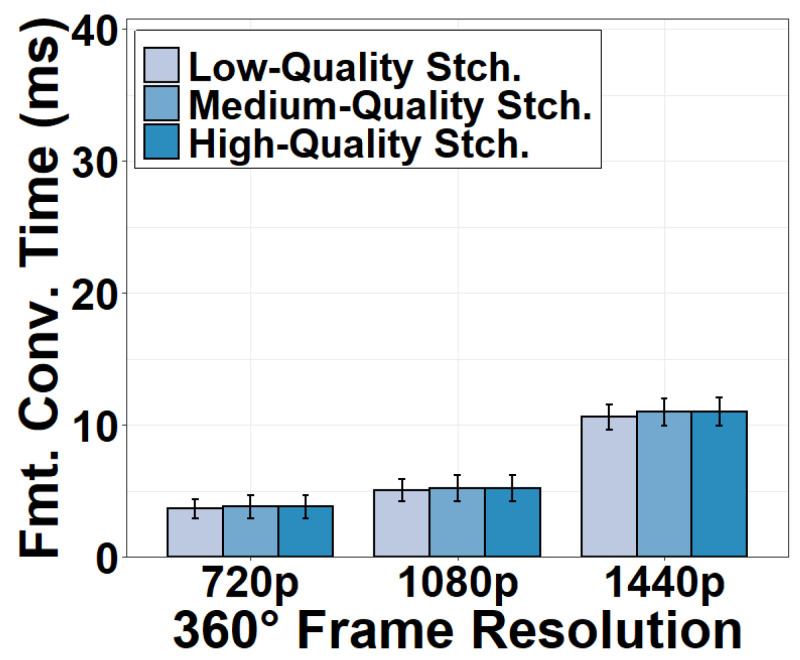
Format conversion time versus stitching options.

After the stitched 360° frame is moved back to the CPU, the video frame in RGB format must be converted to YUV format before encoding. We show time consumption for this format conversion in Figure 7. This task consumes 3.75 ms for a 720p video frame. The time consumption is increased to 10.86 ms for a 1440p frame. This is because video frames with higher resolution have more pixels to convert. We also observe that the stitching quality has a negligible effect on format conversion time because format conversion is primarily affected by the number of pixels to convert rather than the stitching algorithm to execute.

#### 4.2.5. Encoding Task

**Figure 8:**
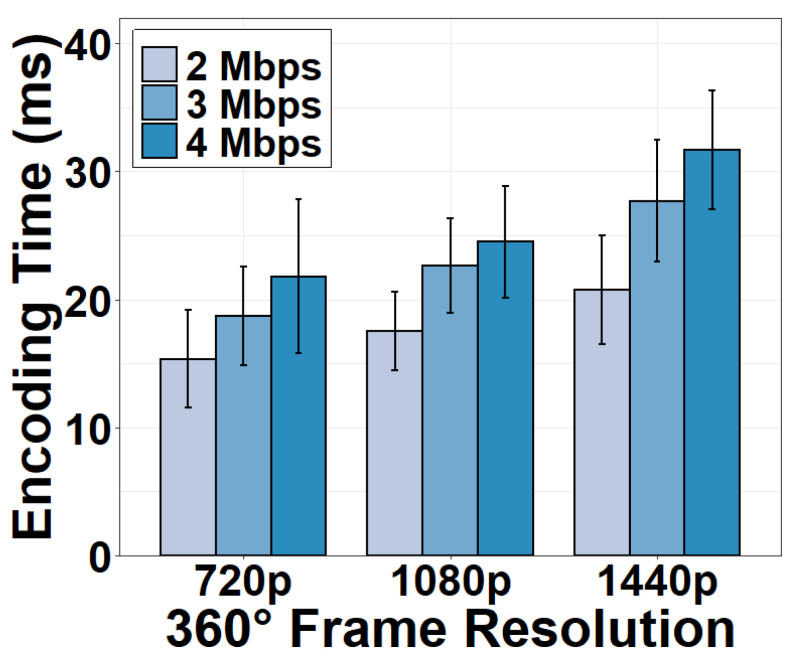
Encoding time under different encoding bitrates.

We show the encoding time under different encoding bitrates and resolutions in Figure 8. As expected, we see that the encoding is a major time-consumer in the camera. It takes 20.74 ms on average to encode a 1440p 360° frame at 2 Mbps. When the resolution is 720p, the encoding time decreases to 15.35 ms because fewer pixels have to be processed and encoded. Another observation is that 1 Mbps decrease of bitrate can lead to 16.68% decrease in the encoding time under the same encoding resolution. A lower bitrate is typically the result of a greater quantization parameter (QP) in encoding. A greater QP results in fewer non-zero values after the quantization, which in turn reduces the time to encode these non-zero values. Given the impacts of encoding time on the camera processing speed, it is critical to strike a tradeoff between the camera frame rate and the encoding quality.

**Figure 9:**
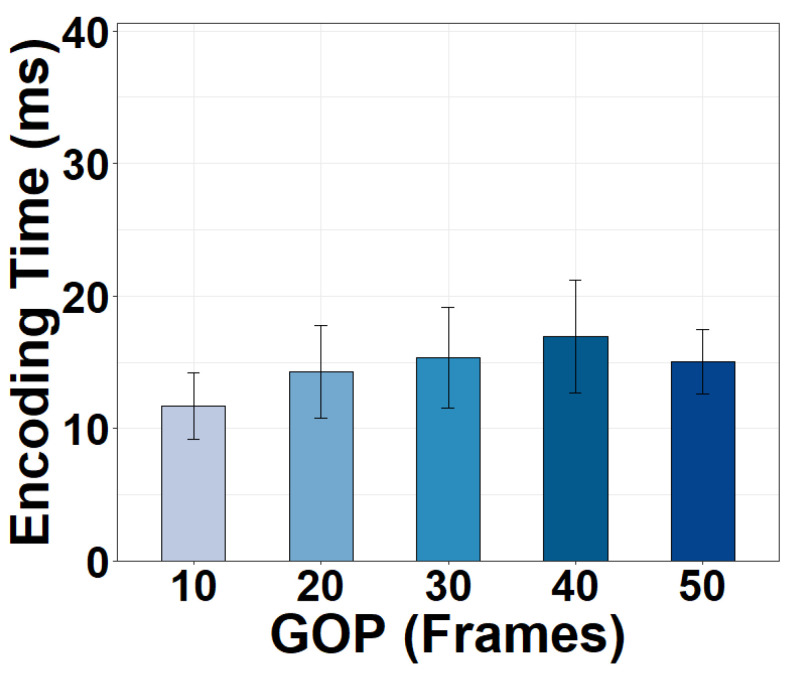
Encoding time of a 720p frame versus GOP.

Moreover, it is interesting to find in Figure 9 that the encoding time is affected by the GOP value. As the length of GOP increases, the encoding time increases but then starts decreasing once the GOP value reaches a certain threshold. A longer GOP requires the encoder to search more frames while calculating the inter-frame residual between the I-frame and other frames. This enlarged search space leads to a longer encoding time. However, today’s encoders automatically insert an I-frame at scene changes if the GOP value is set to be too large. These inserted I-frames would break the long-GOP search and decrease the encoding time. Our measurement implies that the GOP threshold for the automatic I-frame insertion is somewhere from 40 to 50 in modern video encoders.

#### 4.2.6. Impact on Delay Metrics

Our camera is able to achieve 360° camera sensing of 720p videos at 24–30 fps, which is consistent with the performance of state-of-the-art mid-end 360° cameras such as Ricoh Theta S [43]. We find that the camera executes a sequence of tasks for a frame one by one without utilizing parallel processing. Hence, the frame rate of the camera output is simply an inverse of the total time consumption of all tasks in the camera. This can be validated by our observation that the total time consumption for all tasks in the camera is less than 33.3 ms for a 720p frame. More importantly, our measurement suggests that several computing tasks can be optimized to enhance the output quality of the 360° camera. Besides the well-known challenge in encoding, we discover that optimizing the CPU-GPU transfer inside the camera is also important given its noticeable time consumption. On the other hand, there is little scope to improve the stitching task further because the stitching time in current practice is already low. Furthermore, the parameter space of the critical tasks, such as encoding and CPU-GPU transfer, should be explored to balance the camera frame rate and the output video quality. The optimization could potentially enable the support of higher-quality videos that are only offered in high-end cameras or even unavailable in today’s markets.

Note that the tens of milliseconds of camera time consumption will not realistically affect the event-to-eye delay defined in Equation (2). The typical requirement of event-to-eye delay for interactive applications is no more than 200 ms [44]. This requirement can be further relaxed to 4–5 s for non-real-time applications such as event broadcasting and scientific monitoring [45]. Finally, we reiterate that the camera does not affect the start-up delay as defined in Equation (1).

### 4.3. Camera-Server Transmission

**Figure 10:**
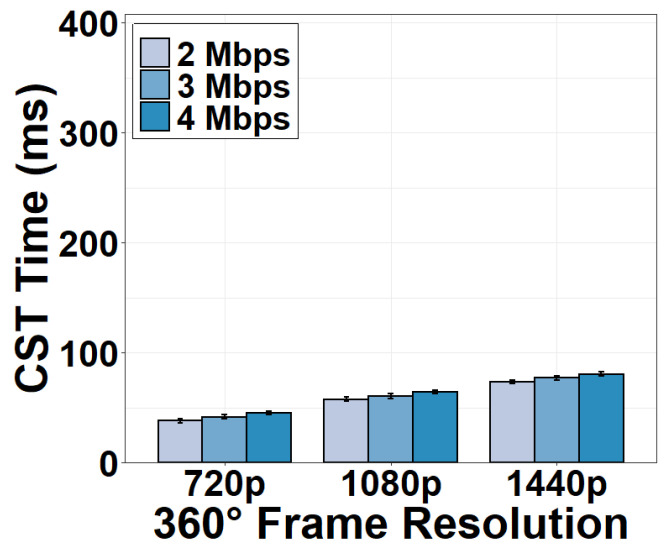
CST time under different encoding bitrates.

We now evaluate the time consumption of the camera-server transmission (CST). We measure the CST time under different encoding bitrates and resolutions of the 360° video sent by the camera. Results in Figure 10 show that the CST time is longer than the time consumption in the camera in general. It is evident that the CST time increases as the encoding bitrate or resolution increases. For example, transmitting a 720p 360° frame at 2 Mbps consumes 37.83 ms and the transmission time increases to as long as 73.23 ms for a 1440p frame.

**Figure 11:**
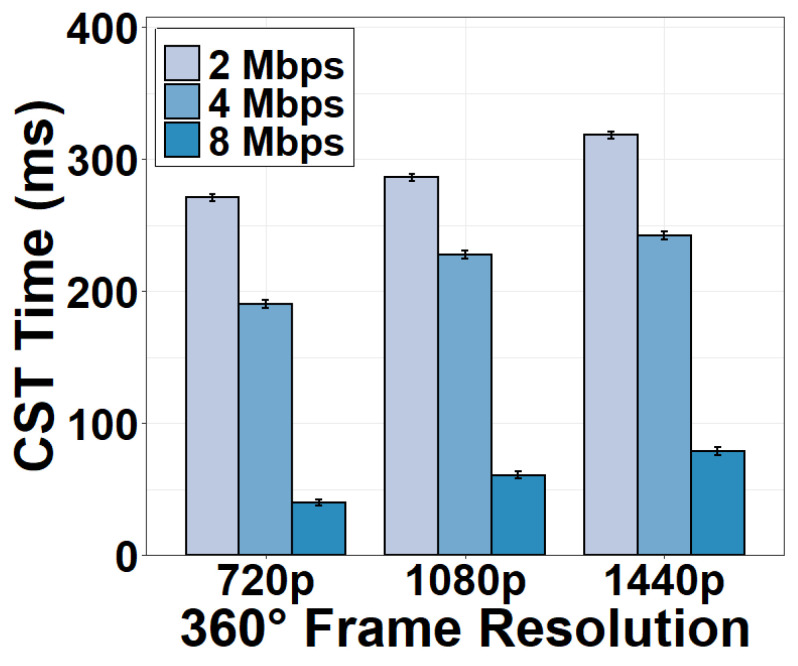
CST time versus upload bandwidth.

In order to evaluate the impact of network conditions on the CST time, we fix the encoding bitrate at 2 Mbps and throttle the upload bandwidth of the CST to 2, 4, and 8 Mbps using *NetLimiter*. We can see from Figure 11 that the CST time dramatically increases when the upload bandwidth is reduced to 2 Mbps. The CST now consumes 270.79 ms for a 720p 360° frame, 286.13 ms for a 1080p frame, and 318.17 ms for a 1440p frame. When the upload bandwidth is 8 Mbps, the CST time is similar to the case of no bandwidth throttling as in Figure 10. This result implies that 8-Mbps upload bandwidth is enough to support the camera to the server transmission.

#### Impacts on Delay Metrics

**Figure 12:**
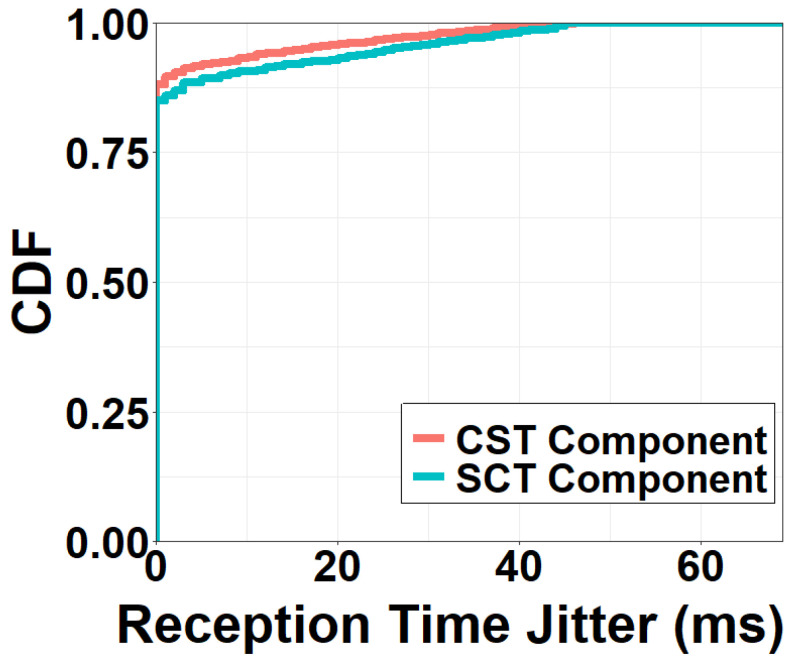
Jitter of packet reception time.

It is important to observe that the time consumption in the CST component generally does not affect the frame rate. This is because the CST component continuously handles video data *packet by packet*. As long as consecutive packets are pushed to the CST component back to back, the output frame rate of the CST component will not change regardless of the processing time of a packet. An exception might occur when the variance of the packet transmission time in the CST component (jitter) is significantly high. Fortunately, we found in Figure 12 that 90% of the packets are received within 2 ms after the reception of their previous packets. Thus, packets flow through the CST component continuously and the exceptionally negative effects on the frame rate are not observed.

As for the start-up delay, the CST component is similar to the camera and does not affect it. Nevertheless, the CST time plays a pivotal role in the event-to-eye delay, especially when streaming high-quality videos in live interactive applications.

Since modern WiFi (802.11ac) has sufficient bandwidth to support a reasonable CST time and stable delay jitter, today’s CST component is ready to support typical camera sensing applications. Future efforts should focus on enhancing the transmission protocols in challenging network conditions for diverse sensing environments.

### 4.4. Video Server

#### 4.4.1. Connection Task

**Figure 13:**
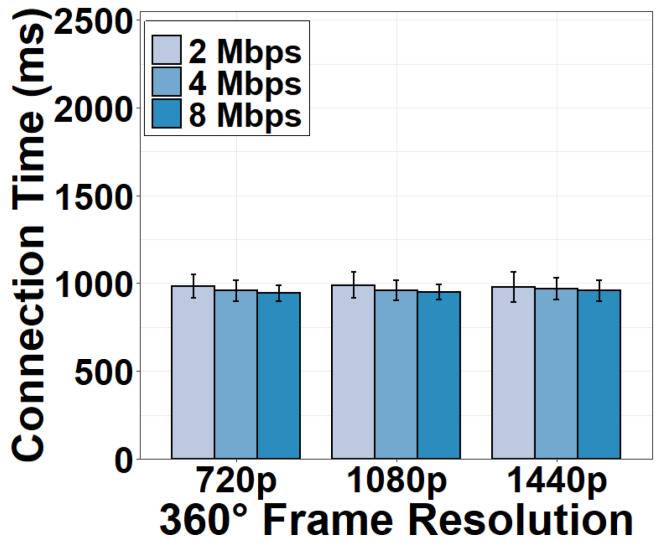
Connection time under different download bandwidths.

Once the 360° video frames received from the camera are sufficient, the server becomes ready to accept a client request. To proceed, the connection task is first conducted. As shown in Figure 13, the time consumption of server-client connection is long, which takes approximately 900 ms. Specifically, the server first spends tens of milliseconds performing a three-way TCP handshake with the client. Then the majority of the connection time (hundreds of milliseconds) is used to prepare the initial HTTP response that accepts the client’s request for the streaming session. To this end, the server must create new threads, initializes data structures for the session management, and registers different handler functions, e.g., ngx_http_request_handler, for accepting the client request. The server then sends the initial HTTP response (excluding video data) to the client. As the HTTP response is not large, we see that a higher download bandwidth does not decrease the connection time significantly.

#### 4.4.2. Metadata Generation and Transmission Task

**Figure 14:**
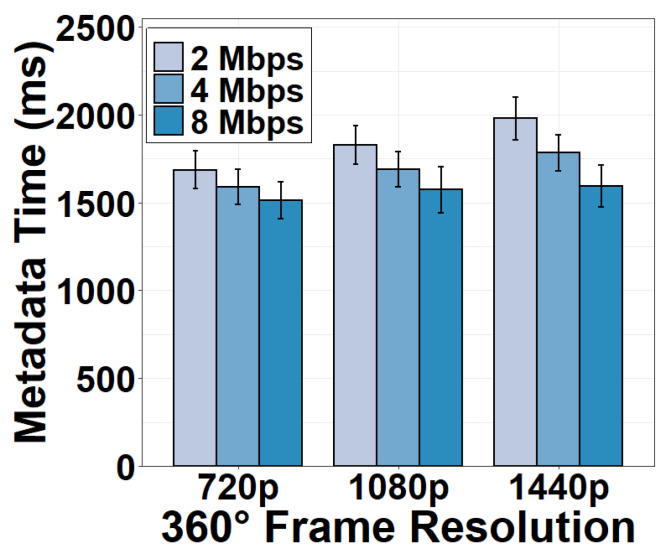
Metadata gen. and tx time under different download bandwidths.

After the HTTP connection is established, the server generates a metadata file and transmits it to the client to convey application-related information. The metadata generation and transmission time under the download bandwidth of 2, 4, and 8 Mbps are shown in Figure 14. The metadata time is long because the server must create and transmit a metadata file detailing the format, encoding, and projection parameters of the 360° video. This procedure involves retrieving video information from the camera through server-camera communication, registering functions and creating data structures on the server, generating live video streaming threads to build the metadata file, and sending it to the client through server-client communication. Since this is not a parallel process, it consumes excessive time to run these processes. The shortest time is 1512.90 ms for a 720p video stream under the download bandwidth of 8 Mbps. We also observe that reducing the download bandwidth does not decrease the metadata time significantly. This implies that metadata generation rather than metadata transmission dominates the time consumption of the metadata task.

#### 4.4.3. Packetization Task

After the connection and metadata tasks, the server is ready to stream video data to the client continuously through the pipelined packetization and transmission. According to our measurement, packetizing video data at the server for video streaming to the client consumes negligible time. The received camera captured frames are packetized as soon as possible without any additional delay. The packetization process is fast itself because the Nginx server leverages pointers to record the locations of the received video data in the server buffer and then directly fetches the video data using the pointers when adding FLV headers to generate HTTP-FlV packets. No memory copy or transfer is needed for the received video data to become a packet.

#### 4.4.4. Impacts on Delay Metrics

As the connection and metadata tasks executed in the server happen before any video frames are pushed into the system pipeline for camera sensing and streaming, they do not affect the frame rate at all. Due to the negligible packetization time, the server also does not affect the end-to-end frame rate in 360° video camera sensing.

However, the large time consumption of the connection and metadata tasks causes a long start-up delay that may discourage users from staying in the video viewing after they click the video URL and initialize the session. The lengthy start-up delay in turn yields an excessive event-to-eye delay. Although the connection and metadata tasks occur only once, video data are accumulated in the server buffer during the session initialization. The video frames arriving at the server later cannot be sent until previous frames are sent out. These frames have to wait for all previous frames, leading to a long event-to-eye delay. This long event-to-eye delay would reduce the responsiveness of the camera sensing applications and degrade the viewing experience. To mitigate the negative effects of long start-up and event-to-eye delay, future efforts can be made to optimize and parallelize the computing workflow and data management in the server in order to minimize the preparation time during the server-client initialization.

### 4.5. Server-Client Transmission

**Figure 15:**
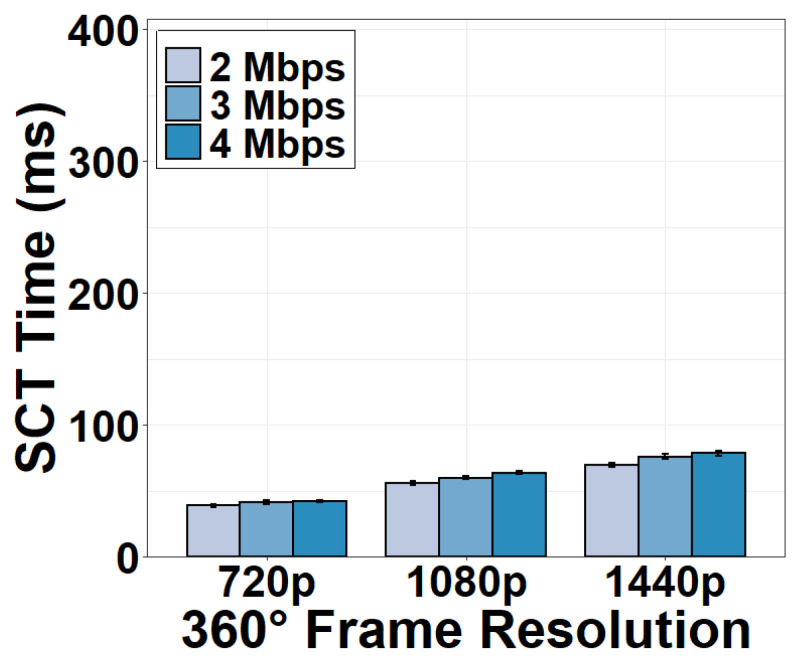
SCT time under different encoding bitrates.

**Figure 16:**
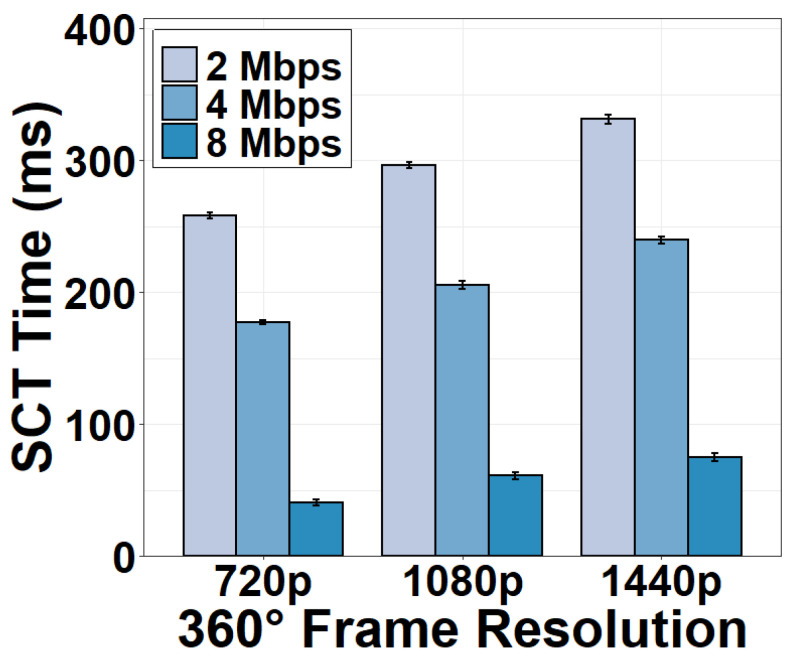
SCT time versus download bandwidth.

Similar to the CST component, we measure the SCT time for streaming a 360° frame from the server to the client. Results in Figure 15 and Figure 16 show that the SCT time is similar to the CST time, taking 41.07 ms for a 720p 360° frame and 74.98 ms for a 1440p frame. The reason for the similar time consumption is that the distance between the camera and the server is almost the same as the distance between the server and the client. Further, both the upload and download bandwidth are sufficiently high to support stable video streaming. As expected, the SCT time also decreases as the video quality degrades and the download bandwidth increases.

#### Impacts on Delay Metrics

Different from the CST component, the SCT component plays a critical role in the start-up delay since the first video frame has to be first streamed to the client before it can be displayed on the client screen. As for the event-to-eye delay and the frame rate, however, the impacts of the SCT are similar to the CST. In particular, the SCT time directly influences the event-to-eye delay. If the streaming time from the server to the client is high, users would have a laggy viewing experience. The impacts of the SCT on the frame rate depend on whether or not packets can be continuously streamed to the client. If the network conditions are unstable, the continuous packet reception in Figure 12 may not hold for the SCT, which could incur a decreased frame rate.

### 4.6. 360° Video Client

#### 4.6.1. Decoding Task

**Figure 17:**
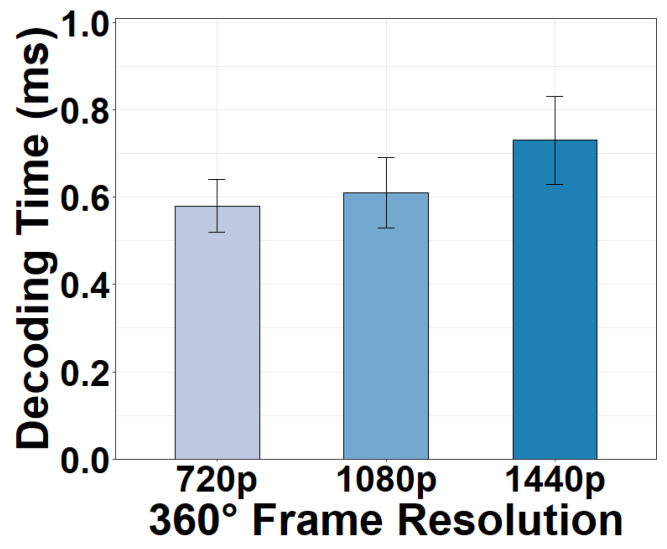
Decoding time versus 360° frame resolutions.

The 360° equirectangular frames received at the client must be first decoded into raw video frames. We show the decoding time of a 360° frame at the client in Figure 17. We observe that the decoding time is negligible, with an average of only 0.62 ms under various resolutions. Indeed, today’s computers utilize hardware-accelerated decoders for video decoding to prevent the large decoding overhead in traditional CPU-based software decoders that requires either longer time or multiple stronger CPUs. This strategy has expedited the complicated decoding process significantly.

#### 4.6.2. Rendering Task

**Figure 18:**
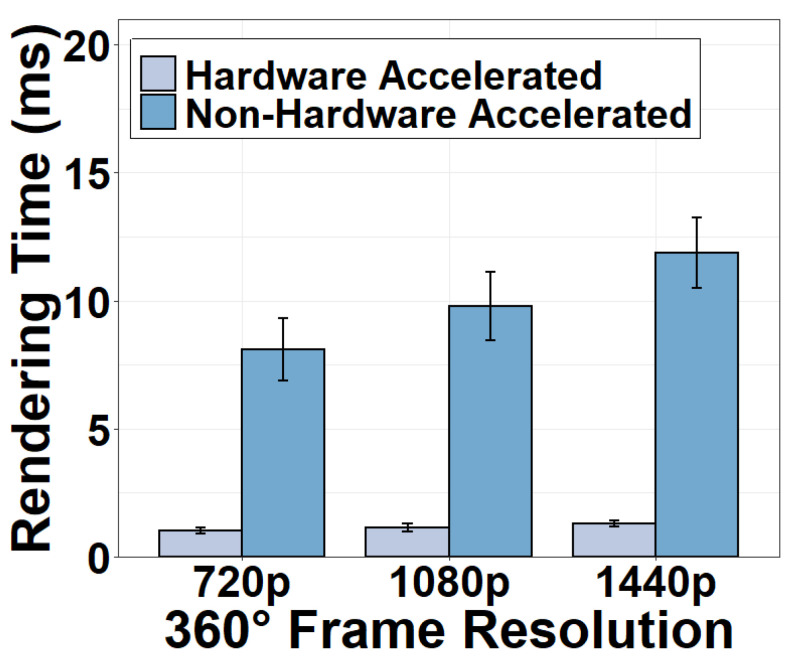
Rendering time of different hardware options.

Figure 18 shows the rendering time of the decoded 360° frame under different hardware configurations. It can be seen from the figure that the rendering time is also negligible and that the hardware acceleration speeds up the rendering. The time consumption for the rendering task includes the time for projecting the equirectangular frame to a spherical frame and rendering the viewport. When GPU-based hardware acceleration is used, the rendering time is only 1.29 ms for a 1440p video frame, an 89.13% decrease from the non-acceleration mode. Such performance enhancement is accomplished by the massively parallel architecture of the GPU processing. It is true that it takes time for video frames to be transferred to the client’s GPU for rendering. However, we point out that this process is much less time-consuming than the CPU-GPU frame transfer in the camera. This is because, at the client, a video frame is fetched from the WiFi module to the GPU through Direct Memory Access without a CPU-GPU transfer.

#### 4.6.3. Display Task

Once the viewport of the 360° frame is rendered, the viewport data are transferred to the display buffer at the client. Then, the client screen refreshes at a pre-defined frequency to display the most recently buffered data. These two steps consist of the display task. We discover that sending data to the display buffer consumes negligible time. Therefore, the display time is actually determined by the refresh frequency. In our system, the screen refreshes at 60 Hz, equivalent to a 16.67 ms display time.

#### 4.6.4. Impact on Delay Metrics

The frame rate of the client output is determined by the inverse of its time consumption because the frames are processed one by one in a non-parallel manner at the client. This is similar to the case of the 360° camera component. Our measurement demonstrates that even with the mandatory 360° video projection and rendering, all client tasks can be completed within a fairly short time to meet the 24–30 fps frame rate. Given the small time consumption at the client, the client’s contribution to the start-up delay and the event-to-eye delay is significantly less than the contribution of the server or SCT component. Hence, we reach a conclusion that the client has minor impacts on the user experience because of its negligible impacts on the three delay metrics. We believe today’s 360° video clients are already well-designed for high-requirement 360° video applications.

### 4.7. More Usage Scenarios

We have so far focused on the measurement result obtained from the Default scenario defined in Section 4.1. In this section, we present the macro-benchmark results under multiple realistic usage scenarios of the 360° video camera sensing system. We aim to compare the measurement results in order to uncover the impacts of the time consumption on delay metrics under different scenarios. The requested video quality for camera sensing is set to be 720p with a bitrate of 2 Mbps. In addition to the Default scenario, we evaluate the following additional usage scenarios of 360° video camera sensing.

- *Simple*: The experiment configuration of this scenario is based on the Default scenario. The only difference is that all the lights in the lab are turned off. The captured content is almost dark except for a laptop screen showing a timer that is used to remind us of the experiment time.
- *Lab Moving*: Building on top of the Default scenario, the Lab Moving scenario involves a moving camera. A person holds the camera rig and walks around the lab while the camera captures content.
- *Street Still*: Instead of fixating the camera in the lab in the Default scenario, Street Still places the camera in a fixed position on the sidewalk of a street in a city center. Other settings remain the same as in the Default scenario.
- *Street Moving*: Based on the Street Still, we configure the Street Moving scenario by having a person holding the camera rig while walking on the street sidewalk.

**Figure 19:**
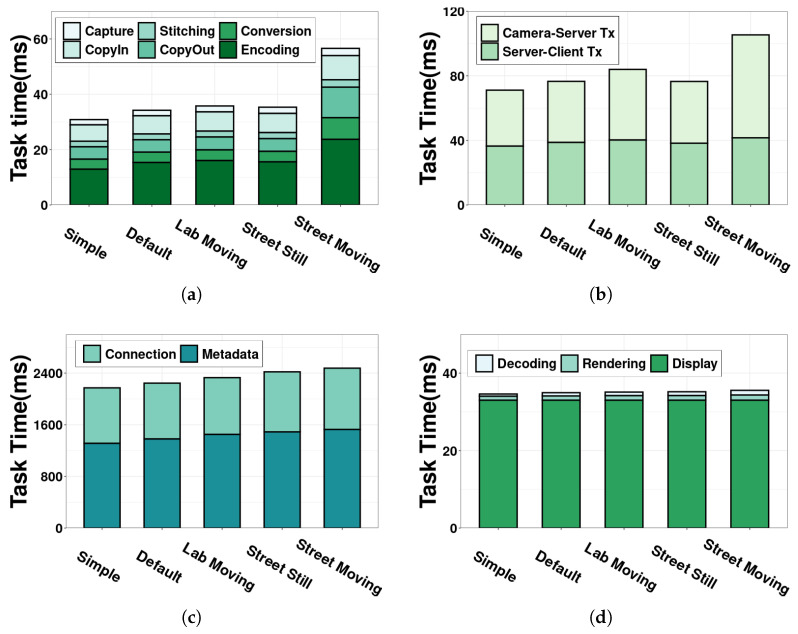
The task-level time breakdown under five usage scenarios for all system components. (**a**) 360° camera; (**b**) CST and SCT; (**c**) Video server; (**d**) 360° video client.

Figure 19a shows the time breakdown of the camera component under different scenarios. We observe that motion has an impact on the time consumption of the camera. Both the Lab Moving and Street Moving scenarios consume more time on the camera than their counterparts in the still scenarios (Default and Street Still) respectively. We notice that the increased time stems mainly from the encoding. For example, it takes 8.12 ms more time for encoding in Street Moving than in Street Still. The reason is that camera motion increases the complexity and diversity of the captured video content, making it more difficult for the encoder to find the redundancy between frames for encoding. It can also be seen that the time increase from Default to Lab Moving is smaller than that from Street Still to Street Moving. This is because the relatively simple background of the lab environment does not introduce much variance to the video content. The time breakdown of other camera tasks in different scenarios presents a similar pattern to the Default scenario. Regarding the impacts on the delay metrics, we find that our insight obtained from the Default scenario still holds in all scenarios. The only difference comes from the camera frame rate. Specifically, the recommended 24–30 fps frame rate can be achieved in all scenarios except for Street Moving. This identifies a possible bottleneck of the moving camera in the wild, which opens a new door for future research in optimizing the computing workflow of 360° camera, especially the encoding in moving scenarios.

The time consumption of CST, SCT and server components is demonstrated in Figure 19b,c. We observe that the time consumption for these three components are higher when the camera is in motion, especially in Street Moving. In addition to the more complicated video content, another reason for the increased time in these components is the decreased bandwidth for video transmission with a moving camera. The effect is evident in the change of the CST time in Figure 19b. It takes more time for a moving camera to transmit data over the uplink to the server. Despite the increased time in some scenarios, we stress how time consumption affects the three delay metrics would not change. One potential issue that needs future research attention is to reduce the CST time in the moving scenarios, which could ensure a low event-to-eye delay. New transmission protocols or bandwidth prediction methods can be designed to improve the CST time.

As for the client, the task time almost remains the same across different scenarios and thus its impacts on the delay metrics do not change. This is because the camera motion would not significantly affect the workflow of the client.

## 5. Cross Validation

To confirm that the results collected by Zeus can be generalized to commercial live 360° video streaming systems and provide insight for system optimization, we perform cross validation by comparing Zeus to a system using commercial products. As it is infeasible to break down the task-level time consumption of black-boxed commercial products, we compare the component-level time consumption.

### 5.1. Experiment Setup

The commercial system uses a Ricoh Theta V as the camera with an Adreno 506 GPU for 360° video processing. An open-source plug-in [46] is installed such that the camera can communicate through WiFi with the server. We use YouTube live streaming service as the server. The YouTube server provides the server URL and the streaming key to the camera. For the commercial client, we use an embedded YouTube client via IFrame APIs and install it on the same laptop used in Zeus.

While obtaining the time breakdown of each task is infeasible on commercial products, we can utilize high-level APIs provided by these products to measure the time consumption on each component. We measure the time consumption of a frame in the Ricoh camera by recording the timestamps when it begins to create a 360° video frame (via scheduleStreaming.setSchedule()) and when the frame starts being transmitted (via rtmpExtend.startSteam()). As for the YouTube server, we measure the time by monitoring its status change via the webpage interface used to configure the camera URL. We utilize the timestamps when a packet is received at the YouTube server and when the streaming starts at the server. Similarly, the time consumption on the YouTube client can be measured by monitoring the buffering status YT.PlayerState. In terms of the frame transmission time for the CST and SCT, we record the timestamps when the sender sends the first packet and when the receiver stops receiving data and then divide this time interval by the number of frames delivered during this period.

### 5.2. Cross-Validation Results

**Figure 20:**
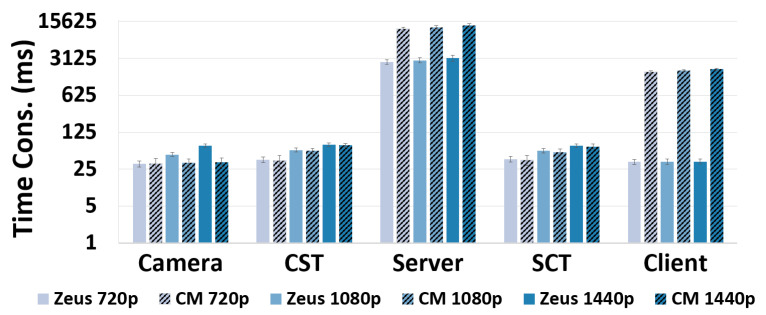
Comparison of component time between Zeus and a commercial system (denoted by CM).

The comparison of the time consumption across the five system components of the two systems is shown in Figure 20. It can be seen that the distribution of the time consumption across system components for Zeus is similar to that for the commercial system. Specifically, the time consumption in the camera, camera-server transmission, and server-client transmission is almost the same, and the server in both systems consumes significant time. We quantify the similarity of component-wise time consumption between the two systems by calculating the Pearson Correlation Coefficient (PCC) [47], the Distance Correlation (DC) [48], and the Cosine Similarity (CS) [49] of the time distribution across the five components. We calculate the correlation using two representative scenarios—Default and Street Moving.

The results in Table 2 demonstrate that the PCC and DC values are greater than 0.98 in both scenarios, indicating that the distribution of time across the five components in the two systems has a strong positive correlation. The high CS value further implies that the 5-element vectors of component time for both systems point in roughly the same direction, leading to the implication that the most time-consuming component of the two systems is the same (the server).

The strong correlation and similarity of the Zeus component-wise measurement results with the commercial 360° video system imply that our results with Zeus are representative of commercial live 360° video streaming systems. Our insight can thus be generalized to minimize the adverse effects on user experience caused by different delay metrics in today’s commercial systems.

**Figure 21:**
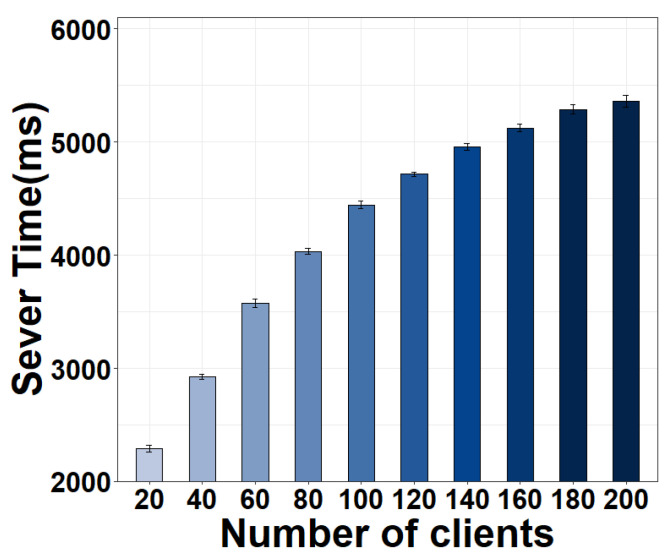
The server time of Zeus increases as the number of clients increases.

In addition to correlation, we observe two minor differences between the two systems. First, it can be seen that the YouTube server consumes more time. This may be because it handles a larger number of clients than the Zeus server. We confirm this effect by running a sensitivity study, where we vary the number of clients sending requests to the Zeus server. The results in Figure 21 show that the Zeus server time does significantly increase as the number of clients increases. Second, we see a longer time at the YouTube client. By scrutinizing the workflow, we find that this is attributed to its larger player buffer (∼1500 ms) compared to Zeus (∼40 ms).

## 6. Conclusions and Future Work

We perform the first in-depth study of delay across the computing tasks in 360° video camera sensing in this paper. We have characterized the three important delay metrics by the time consumption breakdown across the tasks. A prototype Zeus has been developed to measure this relationship and examine the task-level time consumption in various usage scenarios. We finally compare Zeus with commercial systems and validate that our measurement results are representative.

Our findings provide critical insight for improving 360° video camera sensing. First, the bottleneck of a higher frame rate is the 360° camera. Although the space for optimizing the stitching is limited, enhancing the encoding and CPU-GPU transfer may elevate the frame rate to the next level. Second, the server plays a decisive role in meeting the requirement of start-up delay and event-to-eye delay. The existing sever workflow can be optimized to reduce the server time.

In light of these observations, future work should focus on algorithm design in the camera to improve frame rate as well as in the server to shorten the delays and to support multiple clients. After optimizing Zeus in terms of camera frame rate and server delay, it is important to compare Zeus with emerging commercial systems that support 360° video live streaming through advanced cameras, such as Ricoh Theta Z1. A full scale research including extensive experiments are needed to confirm the applicability of the findings in this paper in the context of challenging 4 K 360° video systems. Moreover, since user experience is also determined by the quality of the received 360° videos, it is critical to understand how the delayed and missed packet delivery would affect the distortion and quality of the video frames viewed by the users. A widely-accepted system should provide users with both acceptable latency discussed in this paper as well as satisfactory video quality dictated by 360° image assessment metrics [50,51,52].

## Figures and Tables

**Table 1:** System components and computing tasks in 360° video camera sensing.

Component	Computing Task	Task Definition
Camera	Video capture	Obtain a video frame from each 2D camera lens of the 360° camera and put them in memory
	Copy-in	Transfer the stored 2D video frames from the memory to the GPU
	Stitching	Utilize the GPU to stitch multiple 2D video frames into an equirectangular 360° video frame
	Copy-out	Transfer the 360° video frame from the GPU to the memory
	Format conversion	Use the CPU to convert the stitched RGB frame to the YUV format
	Encoding	Compresses the YUV equirectangular 360° video frame using an H.264 encoder
CST	CST	Deliver data packets of the 360° video frame from the camera to the server
Server	Connection	Establish a connection with the client after a user clicks the live streaming URL
	Metadata Generation	Create a metadata file for the live 360° video and send it to the client
	Packetization	Packetize the received camera data for streaming
SCT	SCT	Transmit data packets of the 360° video frame from the server to the client
Client	Decoding	Convert the received packets to 360° video frames
	Rendering	Project the decoded equirectangula frame into a spherical frame and render the pixels of the
		selected viewport on the spherical frame
	Display	Send viewport data to the display buffer and show the buffered data when screen refreshes

**Table 2:** Correlation of time consumption across five components between Zeus and the commercial system.

Motion	Resolution	PCC	DC	CS
Static	720p	0.989045	0.993842	0.990239
	1080p	0.987980	0.994173	0.990135
	1440p	0.987269	0.994539	0.990206
Moving	720p	0.990334	0.994896	0.992691
	1080p	0.990994	0.995165	0.992799
	1440p	0.992019	0.995811	0.993636

## Data Availability

Not applicable.
